# The Photoprotective Behavior of a Motile Benthic Diatom as Elucidated from the Interplay Between Cell Motility and Physiological Responses to a Light Microgradient Using a Novel Experimental Setup

**DOI:** 10.1007/s00248-024-02354-7

**Published:** 2024-02-13

**Authors:** Jérôme Morelle, Alexandra Bastos, Silja Frankenbach, Jörg C. Frommlet, Douglas A. Campbell, Johann Lavaud, João Serôdio

**Affiliations:** 1 CESAM–Centre for Environmental and Marine Studies and Department of Biology, University of Aveiro, Campus de Santiago, 3810-193 Aveiro, Portugal; 2 Biology Department, Mount Allison University, Sackville, NB Canada; 3 LEMAR-Laboratory of Marine Environmental Sciences, UMR 6539 CNRS, Univ Brest, Ifremer, IRD, Institut Universitaire Européen de La Mer, Technopôle Brest-Iroise, Plouzané, France

**Keywords:** Imaging-PAM fluorometry, Single cell, Microphytobenthos, Photoprotection, Diatoms, Phototaxis

## Abstract

It has long been hypothesized that benthic motile pennate diatoms use phototaxis to optimize photosynthesis and minimize photoinhibitory damage by adjusting their position within vertical light gradients in coastal benthic sediments. However, experimental evidence to test this hypothesis remains inconclusive, mainly due to methodological difficulties in studying cell behavior and photosynthesis over realistic spatial microscale gradients of irradiance and cell position. In this study, a novel experimental approach was developed and used to test the hypothesis of photosynthesis optimization through motility, based on the combination of single-cell in vivo chlorophyll fluorometry and microfluidic chips. The approach allows the concurrent study of behavior and photosynthetic activity of individual cells of the epipelic diatom species *Craspedostauros britannicus* exposed to a light microgradient of realistic dimensions, simulating the irradiance and distance scales of light microgradients in benthic sediments. Following exposure to light, (i) cells explored their light environment before initiating light-directed motility; (ii) cells used motility to lower their light dose, when exposed to the highest light intensities; and (iii) motility was combined with reversible non-photochemical quenching, to allow cells to avoid photoinhibition. The results of this proof-of-concept study not only strongly support the photoprotective nature of photobehavior in the studied species but also revealed considerable variability in how individual cells reacted to a light microgradient. The experimental setup can be readily applied to study motility and photosynthetic light responses of other diatom species or natural assemblages, as well as other photoautotrophic motile microorganisms, broadening the toolset for experimental microbial ecology research.

**Supplementary Information:**

The online version contains supplementary material available at 10.1007/s00248-024-02354-7.

## Introduction

The term microphytobenthos (MPB) is used to describe communities of photoautotrophic cyanobacteria and microalgae, especially diatoms (Bacillariophyta), that form dense biofilms at the sediment surface in many shallow aquatic habitats [1, 2]. MPB is especially important in intertidal systems, where it can represent the main source of primary production in turbid estuaries [3, 4] and mediate sediment matrix properties and processes, including stabilization, carbon cycling, and nutrient fluxes [5]. Sedimentary intertidal zones are extreme environments for primary producers. Due to tidal action, resident organisms are subject to periods of tidal immersion, leading to strong attenuation of the light intensity, fresh sediment deposition, and resuspension, in addition to frost and drought periods depending on their geographic location and seasonality. In addition, irradiance is significantly attenuated by the sediment itself [6, 7], so the photic layer varies from hundreds of micrometers to a few millimeters, depending mainly on sediment grain size [8, 9].

Cellular motility has been seen as a key adaptation to sedimentary environments, particularly in intertidal mudflats, allowing cells to colonize new niches, to rapidly respond to environmental, physical, and chemical microgradients (e.g., light, temperature, salinity, desiccation, pH, nutrients, carbon, and signaling molecules), and to actively exploit resource heterogeneity while avoiding unfavorable regions of the microhabitat [10, 11]. In cohesive sediment, MPB is dominated by raphid, pennate, epipelic diatoms, species that are capable of oriented motility within the matrix of fine sediment particles [11–15], with a rhythmic pattern that repeats according to low tide and photoperiod cycles [11]. These diatoms move through gliding, associated with forceful discharge of exopolysaccharides through the raphe system [16], a unique trait among diatoms [17] that has been hypothesized to have conferred a critical adaptive advantage to pennate species, explaining their evolutionary success and fast diversification [10, 13].

In nutrient-rich intertidal sediments, light is the main factor limiting photosynthesis and growth of MPB [3, 18–20]. Due to sediment mixing, e.g., induced by tidal currents, MPB cells may be dislocated into sediment layers where the light received is either too low or too high for optimal photosynthesis. When tidal emersion coincides with midday, MPB diatoms can be exposed to a light excessing 2000 µmol photons m^−2^ s^−1^ [3, 21], potentially leading to photoinhibition [22, 23]. A long-standing paradigm, referred to “behavioral photoprotective hypothesis,” stipulates that epipelic diatoms use light-driven motility (phototaxis) in the form of vertical migration within the photic zone of the sediment to adjust their position along the light gradient, optimizing photosynthesis while avoiding photoinhibitory light levels [11].

Benthic diatoms photobehavior and its interplay with physiological processes, such as photoprotection and photoacclimation, have attracted considerable scientific attention over almost four decades [24–30]. The idea that light-oriented motility of epipelic diatoms is a suitable photoregulatory mechanism is strongly supported by the restricted dimensions of the sedimentary photic zone [4, 8, 24, 31] and by the relatively high speeds of epipelic diatoms, up to several tens of micrometers per second [32–35], enabling them to cross the entire photic zone in short, biologically relevant periods. Moreover, in addition to regular time- and tide-synchronized migrations, epipelic diatoms exhibit positive phototaxis under low light levels and negative phototaxis (downward migration) under high light levels, with the migration rate depending on light intensity [26, 36–38].

However, despite the large body of work in support of the behavioral photoprotective hypothesis, the available data does not yet prove the photoprotective role of motility because an unequivocal relationship between diatom phototaxis and an actual decrease in PSII photoinactivation had not been demonstrated [30]. This has been primarily due to methodological limitations in the study of cells migrating inside sediment matrices. Most studies investigating MPB photosynthesis have been based on probe measurements performed from the surface of the sediment [14, 39, 40]. While this approach is suitable to measure biomass and photosynthetic performance of cells present in the photic zone, it cannot be used to assess the physiological benefit of negative phototaxis under high light levels, as cells that migrate below the photic zone are inaccessible to such surface measurements. The only study that investigated the effects of photobehavior on photophysiology was performed using unrealistically wide spatial gradients of irradiance, over a centimeter scale [41]. In addition, motility varies significantly between species and from cell to cell, even in a monogenic population [37]. The gliding movement was shown to be related to the form of the raphe system, which is species-specific [42], and important changes in cell-specific speed and acceleration were shown at the scale of seconds related to jerky diatom motion [43]. Due to these specificities, it has been reported that diatom population motility, speed, and acceleration showed considerable inter-cell variation even in monogenic cultures [44], so that population averages are unrepresentative of actual behaviors.

Therefore, the question of whether, and how much, diatom motility alleviates photoinhibition is still unanswered, and the hypothesis of a photoprotective behavior has not been directly tested. To fill this knowledge gap, it is necessary to investigate these processes under realistic spatial gradients of light intensity, at the single-cell level, while controlling relevant forcing variables. In this study, we describe a new experimental setup that allows the exposure of diatom cells to light microgradients from darkness to full surface irradiance, applied over a millimeter spatial scale, generated inside microfluidic chips, while simultaneously monitoring cell movement and photosynthetic activity in real time and non-invasively, using in vivo chlorophyll fluorescence microscopy imaging. With the primary aim of a proof of concept, we demonstrate the use of the system by testing the behavioral photoprotective hypothesis with the raphid pennate diatom *Craspedostauros britannicus* [45].

## Material and Methods

### Light Microgradient

To investigate the photoprotective role of motility at the individual cell level, a setup was developed to enable the concurrent characterization of the photobehavior and the photophysiology of specific diatom cells exposed to a millimeter-scale gradient of photosynthetically photons flux densities (PPFD).

**Fig. 1:**
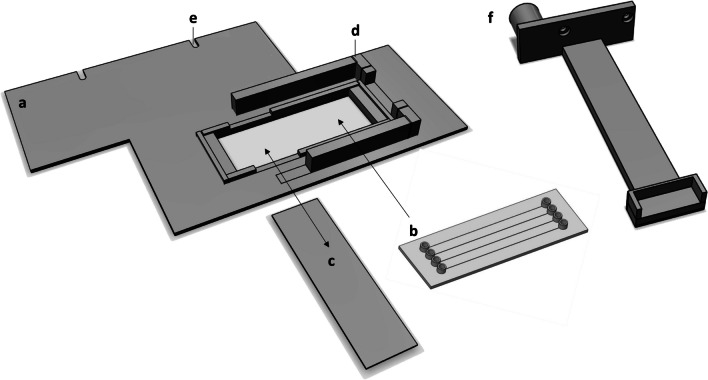
Schematic of the 3D-printed holders used in this study. The sample holder (a) comprises a specific space for positioning the microfluidic chip (b), rails allowing the opaque slide (c) to be easily inserted and removed, walls (d) for positioning the quantum sensor directly above the channels of the microfluidic chip, and notches (e) to fix the holder to the microscope stage. The quantum-sensor holder (f) was designed to connect the quantum sensor with a motorized actuator allowing accurate PPFD gradient measurements. The schematic of the microfluidic chip (b) was obtained from the LabSmith website describing the microfluidic chip used (Fluidic 138; ChipShop, Jena, Germany)

The light microgradient was generated using the illumination system of an upright light microscope (Axio Scope A1, Zeiss, Göttingen), with light incident from below, inside a straight channel of a transparent microfluidic chip, made of polymethyl methacrylate (PMMA) (Fluidic 138; ChipShop, Jena, Germany) (Fig. 1, item b). Each of the four channels per chip had dimensions of 1000 × 200 × 5850 µm (width × height × length) and a 175-µm-thick lid. The motility and photophysiological responses of individual cells inside the microfluidic channel and along the microgradient were recorded from above, through the microscope objectives.

The microscope light source was the halogen lamp of the microscope (OSRAM 50W 12 V) upon which two optical filters were applied: a blue filter (1,101,001,900,355; Motic Deutschland GmbH, Wetzlar, Germany), to balance the blue vs. red regions of the visible spectrum, and an UV/IR cut-off filter 390–690 nm (49–809, Edmund Optics Ltd, York, UK) to eliminate thermal infrared wavelengths and limit the heating of the samples inside the channel. The final light spectrum provided in supplementary information (Online Resource 1) was assessed using a FLAMES-XR1-ES spectrometer (Ocean Insight, Ostfildern, Germany).

**Fig. 2:**
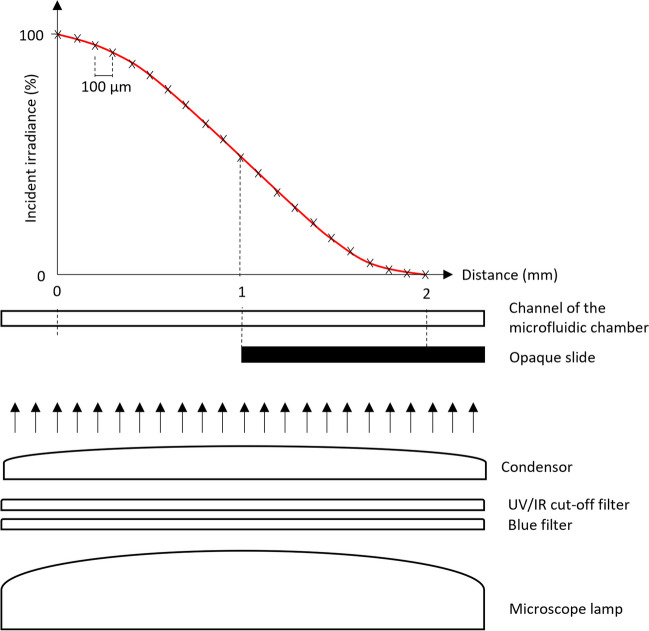
Creation of the light microgradient. The incident light was provided by the microscope halogen lamp. An opaque slide was positioned under the microfluidic chip, resulting in a well-defined gradient of photosynthetic photon flux densities (PPFD) projected onto the channel of the microfluidic chip. The microscope condenser was adjusted to create the narrowest gradient possible, of around 2 mm from 100 to 0% irradiance. The PPFD gradient was measured every 100 µm using a quantum sensor positioned using a motorized actuator and could be described by fitting a hyperbolic tangent curve (*y* = tan(*a*(*x* − *b*) + *c*) × *d*; red line)

To generate the light microgradient over a realistic millimeter-distance scale, an opaque plastic slide was placed directly below the microfluidic chip, with its edge positioned halfway through the microscope view field, allowing a narrow and progressive distribution of the light along the channel. The scattering of upwelling light created a well-defined light gradient whose increase followed the shape of a hyperbolic tangent curve (Fig. 2). The intensity of the microscope light source and the position of the microscope condenser were adjusted to obtain a light gradient over a distance as close as possible to that experienced by benthic diatoms in sediments, spanning 1 to 2 mm [4, 8, 40] starting at a maximum of about 2500 and declining to near 0 µmol photons m^−2^ s^−1^ of PPFD. The highest PPFD value was chosen to represent the maximum values experienced by benthic diatoms under natural conditions at the sediment surface and under direct sunlight [46]. The PPFD measurements at the top of the channels were performed using a quantum sensor (MC-MQS/OVV Microscopy Quantum Sensor; Heinz Waltz GmbH, Germany) calibrated for the microscope light by comparison with a calibrated photosynthetic active radiation (PAR) sensor (Mini Quantum Sensor LS-C, Heinz Waltz GmbH, Germany). To measure the light distribution along the microgradient, a motorized actuator (Motor Mike, Oriel Corporation, Stamford, Connecticut) was coupled with the quantum sensor and positioned to the side of the microscope using a 3D printed holder (Fig. 1, item f). The actuator was connected to an Encoder Mike controller (Model 18,011; Oriel Corporation, Stamford, Connecticut), allowing PPFD measurements every 100 µm along the channel. PPFD measurements were performed before each experiment, after filling the channels with growth medium.

The chip was positioned onto the microscope stage in a way that the linear channel was aligned with the light microgradient. To allow for the precise positioning of the microfluidic chip on the projected light gradient, a chip holder was 3D-printed (Fig. 1, item a). This holder also allowed precise alignment of the quantum sensor along each of the four channels and the opaque slide (Fig. 1, item c) to be easily inserted and removed below the chip. The flexible positioning of the opaque slide enabled the comprehensive observation of cell locations along the entire gradient upon temporary removal of the opaque slide for brief observations of ~ 20 s. The holders were designed using the 123D Design software (Autodesk, Mill Valley, USA) and 3D-printed using black PLA [47].

The motile behavior of the cells along the entire light microgradient was observed using a 10 × objective, providing a view field of 2500 µm in diameter. The photophysiological responses of individual cells were assessed using a Microscopy-Imaging-PAM fluorometer (Heinz Waltz GmbH, Germany), comprising the microscope and a camera IMAG-K6 CCD (2/3″ chip with 1392 × 1040 pixels primary resolution and 4-pixel-binning). This system allows measurement of in vivo chlorophyll fluorescence parameters based on the analysis of images of individual cells. The fluorometer was used in a darkened room to reduce the influence of ambient actinic light, at room temperature, and was controlled by the ImagingWin V2.47 software (Heinz Waltz GmbH).

### Biological Material

The species chosen for this study was the benthic diatom *Craspedostauros britannicus* [45] (Online Resource 2) because of its high motility, with cell speed ranging between 1.2 and 3.5 µm s^−1^, corresponding to 4–12 mm h^−1^, and minimal formation of cell aggregates, ensuring that the movement of individual cells was not impaired by the other cells nor affected by population suspension density. In addition, this species has already been used to study diatom motility [30]. The studied strain of this species (Collection no. NCC195-06–02) was obtained from the Nantes Culture Collection-France (NCC) (http://ncc.univ-nantes.fr/) and was grown in an incubator (Panasonic Versatile Environmental Test Chamber MLR-352, Kadoma, Japan) in 100-mL Erlenmeyer flasks with autoclaved seawater enriched in *f*/2 medium and silica, refreshed every 2 weeks [48]. The incubator was set to a temperature of 18 °C with a PPFD of 20 µmol photons m^−2^ s^−1^, and a diurnal cycle of 12 h light:12 h dark. The culture biomass was measured by transferring 200 µL from the Erlenmeyer into a black 96 well plate with a transparent bottom (Molecular Probes® 96-well microplates, ThermoFisher Scientific, Waltham, USA) and then measuring the Optical Density (OD) at 440 nm using a plate reader (Synergy H1, Biotek, Winooski, USA).

### Experiment Outline

A microfluidic channel was filled with 20 µL of culture (OD_440_ = 0.15), and the wells of the channel were covered with Parafilm (Pechiney, Chicago, USA) to prevent evaporation and a consequent increase in salinity during the experiments. After a dark acclimation of 15 min, to allow the oxidation of the PSII reaction centers and attachment of the cells to the bottom of the channels, each cell located over the entire light microgradient area was excited by a non-actinic modulated measuring light to determine the minimal level of fluorescence (*F*_0_) and by a high-intensity saturation pulse to determine the maximum fluorescence (*F*_m_), allowing to calculate the initial maximal quantum yield of PSII (*F*_v_/*F*_m_ = (*F*_m_ − *F*_0_)/*F*_m_). Both the measuring and actinic lights were blue (450 nm).

The positions of individual cells over this area were obtained by recording an image using a digital camera featuring a 64-megapixel 1/1.73″ sensor with 0.8 μm pixels (ƒ/1.9 aperture, 26-mm wide-angle lens). For this, an additional neutral filter was added to the light source allowing the decrease of the maximal PPFD to 10 µmol photons m^−2^ s^−1^ over the entire area, after removal of the opaque slide. Then, the opaque slide was inserted, the additional filter was removed, and the motile behavioral upon light exposure was monitored over the course of 32 min. Throughout the light exposure period, the cell positions were recorded in the visible portion of the light microgradient by capturing an image every minute over the course of 32 min. The location of cells over the entire light gradient area was recorded every 10 min, in less than 30 s, by removing the opaque slide and placing the additional neutral filter to the light source. Directly after the 32-min light exposure period, the opaque slide was removed, and the location of cells over the entire light gradient area was recorded. Then, prior to any dark relaxation period, each cell within the view field was excited by a non-actinic modulated measuring light to determine the steady state fluorescence (*F*_s_) and by a high-intensity saturation pulse to determine *F*_m_ʹ, to calculate the effective quantum yield of PSII (Δ*F*/*F*_m_ʹ = (*F*_m_ʹ − *F*_s_)/*F*_m_ʹ). The recovery of the maximal quantum yield of PSII (*F*_v_/*F*_m_) for each cell within the view field was measured after 10-min intervals in the dark, over 30 min. To estimate the light-induced photoprotection through thermal dissipation of energy, non-photochemical quenching (NPQ) index was calculated based on the fluorescence values (*Y*(NPQ) = *F*_s_/*F*_m_ʹ − *F*_s_/*F*_m_).

### Control Experiments

To confirm that no factor other than the light microgradient was influencing the observed behavior and photophysiology, two control experiments were carried out. The same experimental outline was followed without the opaque slide under flat irradiance across the entire area. Thus, low light (LL; PPFD of 23.2 ± 1.6 µmol photons m^−2^ s^−1^) and high light (HL; PPFD of 2231.6 ± 209.7 µmol photons m^−2^ s^−1^) were applied for 32 min, followed by a 30-min dark recovery period. For both light levels, the light field generated by the microscope lamp was not completely homogeneous, with PPFD varying along the studied area by 15% from a maximum at the center.

A third control experience was carried out to investigate the role of motility on the photophysiology using the diatom motility inhibitor latrunculin (Lat) A. Therefore, 1 mL of cell culture was exposed to 1 mM of Lat A (Sigma-Aldrich, Saint-Louis, USA) to immobilize all cells along the experiment [8, 39, 46, 49]. After 45 min of pre-incubation time with Lat A in the dark, exposure to the light microgradient and subsequent dark recovery was followed as described in the experiment outline.

### Data Treatment

#### Initial Cell Population Parameters

The physiological responses to light of the cell population were assessed before each experiment by measuring steady state light-response curves (SSLCs) [50] of the relative electron transport rate of PSII (rETR), using a Multi-Color PAM fluorometer, controlled by the PamWin V3.12w software (Heinz Walz GmbH). Briefly, 1250 µL of culture was used in a 1-cm path glass cuvette, and fluorescence was measured using an ED-101US/MD optical unit, coupled to a magnetic stirrer (PHYTO-MS Miniature Magnetic Stirrer, Walz). In the MCP-D detector unit, a RG 665 long pass filter (> 650 nm, 3 mm RG665, Schott) was used. The fluorometer was zeroed using the growth medium as a blank. The measuring light was blue (440 nm), and the actinic light and saturating pulses were white. After 15 min of dark relaxation, the SSLCs were generated by exposing the cell suspensions to 9 increasing PPFD levels (*E*), from 0 to 2197 μmol photons m^−2^ s^−1^, for 120 s under each light level. The relative electron transport rates of PSII was calculated as rETR = (*F*_m_ʹ − *F*_s_)/*F*_m_ʹ × *E* [51], and rETR vs. *E* curves were described by fitting the model of Eilers and Peeters [52], estimating the following parameters: *α* (maximum light efficiency use; µmol photons^−1^ m^2^ s), rETR_max_ (maximum rETR; relative unit), *E*_opt_ (optimal light for photosynthesis; μmol photons m^−2^ s^−1^), and *E*_k_ (the light-saturation coefficient; μmol photons m^−2^ s^−1^).

#### Image Analysis

**Fig. 3:**
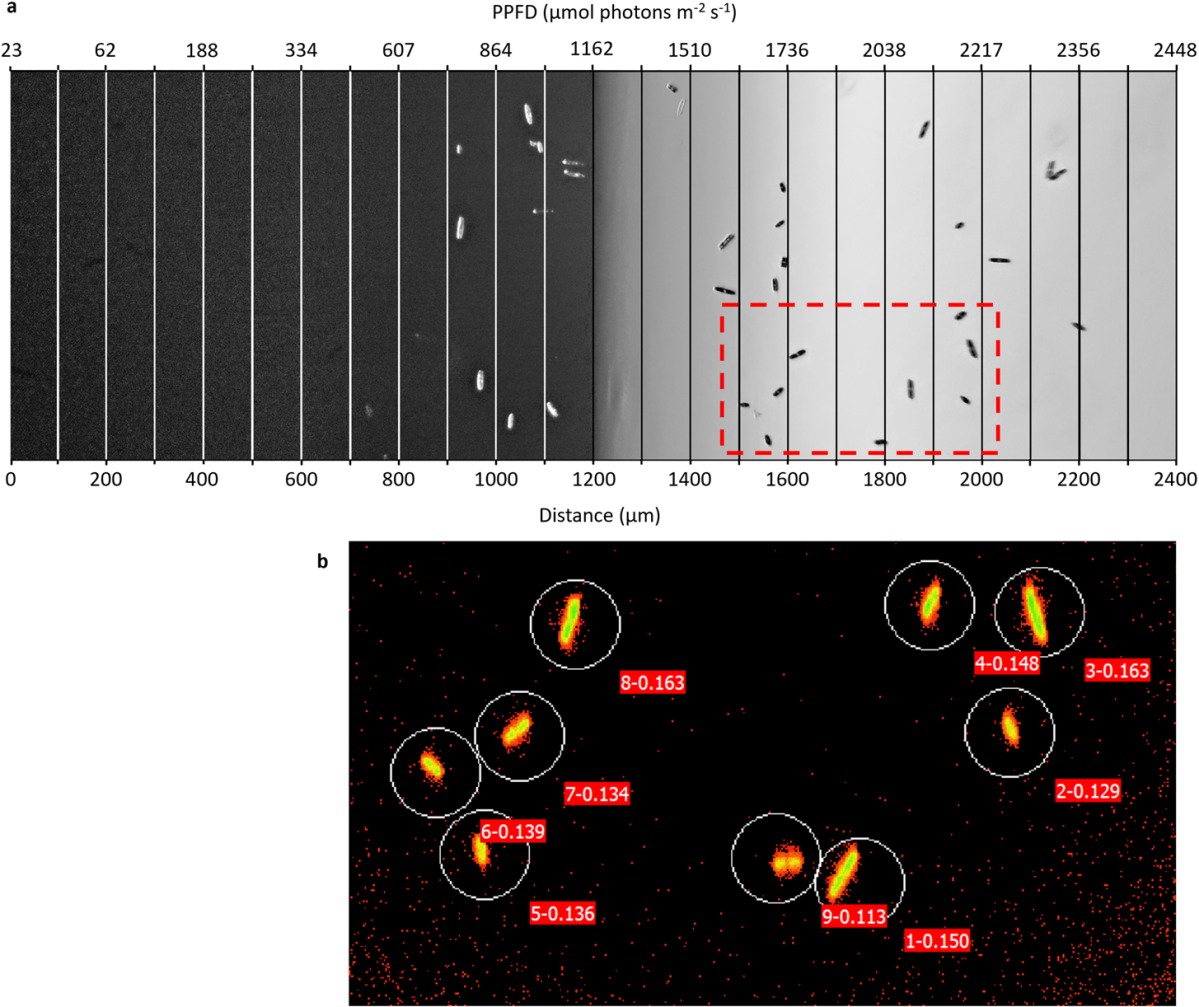
Representative example of **a** an image showing cell distribution, as produced every minute over the experiment, along the light gradient. Overlaid grid indicates the correspondence between the distance along the channel and the photosynthetic photon flux densities (PPFD) measured which could be described by fitting a hyperbolic tangent curve (Fig. 2). Note: As indicated by the PPFD values, the whole channel (0–2400 µm) was illuminated with a gradient of increasing PPFD (23–2448 µmol photons m^−2^ s^−1^). However, the camera exposure settings were adjusted to best capture diatom cells, resulting in a gray balance and a dark appearance of the left-hand side of the image not corresponding to the real PPFD at each location. **b** An image obtained using the Imaging-PAM software showing the photophysiology of individual cells. Circles represent areas of interest labelled 1 to 9, while decimal values represent *F*_m_. The imaging-PAM images were matched to the cell distribution image to compare the measured fluorescence levels and the PPFD received by each individual cell at the time of measurement

The different photosynthetic photon flux densities (PPFD) distributed along the gradient were marked on images by adding a grid with boxes 100 µm wide, corresponding to the distance between PPFD measurements (Fig. 3a). The position of each cell was registered in each recorded image every 1 min or every 10 min for cells obscured by the opaque slide. The following hyperbolic tangent equation (Eq. 1) was fitted to the PPFD with distance values on each image allowing determination of the PPFD experienced $(y)$ at each cell position $(x)$.

1 $$y={\text{tan}}\left(a\left(x-b\right)+c\right)\times d$$

The distance covered by each cell within a certain period of time was determined by measuring the distance between the cell apical point between the successive images. The time separating two images was then used to estimate the net displacement of the cells over each time interval. The accumulated light dose over each time-interval was calculated based on the mean PPFD experienced between two consecutive images, multiplied by the time elapsed between the images. The cumulative light dose received by each cell during the light exposure period was computed by summing up all the individual light doses accrued over each time interval across the entire timeline.

#### Imaging-PAM Records

Because the fluorescence images covered only a fraction of the view field (800 µm length × 600 µm height) and thus only a fraction of the light gradient, 6 to 8 images were captured to cover the entire light gradient. For each fluorescence measurement (i.e., initial *F*_v_/*F*_m_, Δ*F*/*F*_m_ʹ after 32 min of light exposure, and *F*_v_/*F*_m_ over 10, 20, and 30 min in the dark), the fluorescence images were superimposed on the images recorded at the same time. The fluorescence measurements were thus matched to the corresponding cells distributed along the light gradient (Fig. 3). Absolute fluorescence signal values below 0.04 were not considered, following the manufacturer’s instructions (Walz, Effeltrich, Germany).

#### Statistical Analyses

Statistical tests were performed using the software Sigma-Plot V12. To perform statistical tests, the individual cell data were binned into five irradiance classes: 0–500, 500–1000, 1000–1500, 1500–2000, and 2000–2500 µmol photons m^−2^ s^−1^, corresponding to their position on the light gradient. The Shapiro–Wilk normality test was used to determine if the data distribution significantly deviated from a normal distribution, and the Bartlett test was used to assess homogeneity of variances in the datasets. When those tests were validated, significance of the differences between groups of values for each light range was assessed through a one-way ANOVA (aov) followed by a TukeyHSD test. When normality and variance conditions were not met, a Kruskal–Wallis (kw) followed by a pairwise test (Dunn’s method) was performed. Correlations between the different studied variables were assessed using the Pearson correlation analysis.

## Results

### Initial Photophysiological Conditions

The photophysiological parameters measured on suspensions of the *Craspedostauros britannicus* culture used for the experiments showed high quantum efficiency of PSII photochemistry, with *F*_v_/*F*_m_ = 0.590 ± 0.02. The SSLCs measured before each experiment showed similar shapes with *α* = 0.53 ± 0.06 µmol photons^−1^ m^2^ s, rETR_m_ = 136.5 ± 15.5, and *E*_k_ = 259 ± 32 µmol photons m^−2^ s^−1^. *E*_opt_ varied between 879.4 and 1402.6 µmol photons m^−2^ s^−1^ across the samples, with an average of 1084.3 ± 223.4 µmol photons m^−2^ s^−1^.

The initial *F*_v_/*F*_m_ measured on individual cells also confirmed the high quantum efficiency of PSII photochemistry, with values between 0.552 and 0.705 and an average of 0.638 ± 0.04 (number of measured cells (*n*) = 40). High initial *F*_v_/*F*_m_ values were also observed for the control experiments, reaching 0.654 ± 0.07 (*n* = 31) and 0.656 ± 0.09 (*n* = 57) for the LL and HL conditions, respectively. For the Lat A experiment, the initial *F*_v_/*F*_m_ values reached 0.570 ± 0.08 (*n* = 114).

### Cell Behavior During Exposure to the Light Gradient

**Fig. 4:**
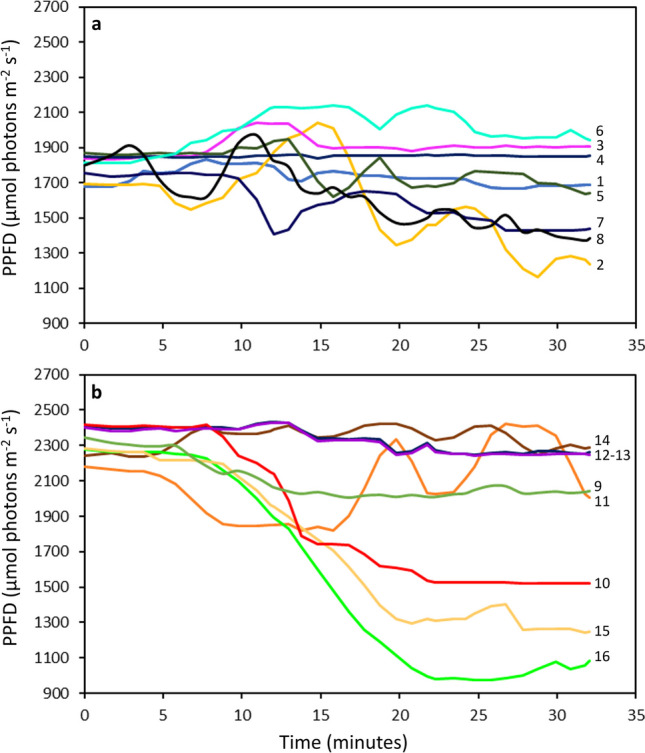
Variation over time of the different photosynthetic photon flux densities (PPFD) experienced by each individual cell during the exposure period. **a** Cells initially exposed to 1500–2000 µmol photons m^−2^ s^−1^; **b** cells initially exposed to 2000–2500 µmol photons m^−2^ s.^−1^. The cells were numbered to facilitate comparison with this figure and Fig. 5, Online Resource 3, and Online Resource 4

The main experiment started with 40 diatom cells within the light microgradient. Of these, 18 cells (16 motile cells and two that did not move) were monitored throughout the entire experiment. The length of those monitored cells varied between 18 and 50 µm. Of the motile cells, 8 were initially located in the region exposed to 1500–2000 µmol photons m^−2^ s^−1^ (Fig. 4a; Online Resource 3) while 8 were initially located in the region exposed to 2000–2500 µmol photons m^−2^ s^−1^ (Fig. 4b; Online Resource 4a-g). The two non-motile cells were initially located in the 948–1126 µmol photons m^−2^ s^−1^ region (Online Resource 4 h).

**Fig. 5:**
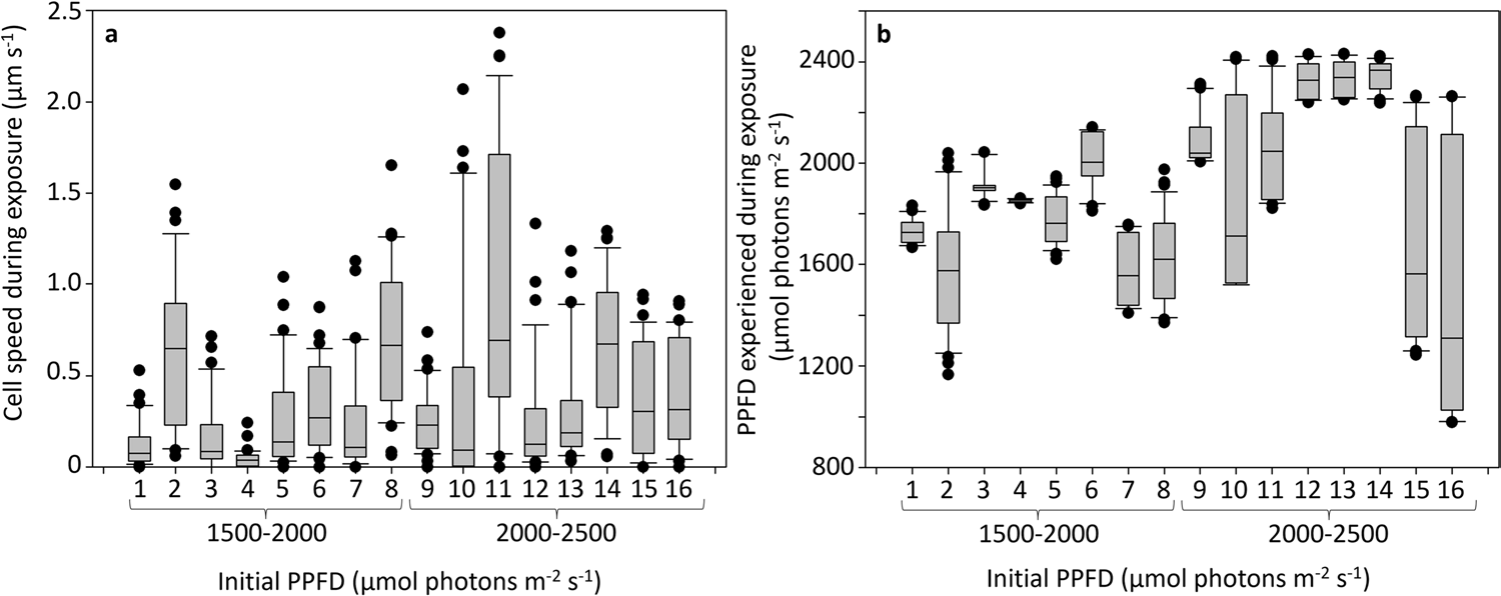
Variation of **a** the speed and **b **the different photosynthetic photon flux densities (PPFD) experienced by the individual cells, initially exposed to 1500–2000 and 2000–2500 µmol photons m ^−2 ^S^−1^, during the exposure period. Individual cell speed and experienced PPFD during exposure were determined according to the cell position and distance covered at each 1-min interval, over 32 min. Each box represents the interquartile range (IQR), corresponding to the range between the first and the third quartile, for the dataset of each individual cell. The line inside the box represents the median while the whiskers indicate the range of the data (minimum and maximum values within 1.5 times the IQR), excluding outliers. Outliers, or extreme values, are represented by the individual dots beyond the whiskers

During the first 5 min of light exposure to light, the cells did not move significantly and largely remained in the region where they had been initially deposited. During this period, cells experienced an average variation in PPFD of 40 µmol photons m^−2^ s^−1^ (Fig. 4). After this initial 5-min period, cells began to move, thereby experiencing other PPFD. The cells exhibited various patterns of motility behavior including circular movements (e.g., Online Resource 3b, 3f, 4c), linear trajectories (e.g., Online Resource 4a, 4b, 4 g), back and forth movements (e.g., Online Resource 3a, 3e), and sometimes with one pattern following another (e.g., Online Resource 3 h, 4e). During this phase, the speed of the cells (net displacement over time) reached up to 2.37 µm s^−1^ (cell n°11), but different cells showed substantial variability in speed (Fig. 5a). Over the 32 min of light exposure, the distance covered by the cells varied between 147 µm (cell n°4) and 3463 µm (cell n°11), with an average of 1347 ± 860 µm. Nevertheless, it is important to note that the net displacement distance covered along the light gradient was actually lower, averaging 428 ± 288 µm per cell, with a maximum of 993 µm (cell n°16). This was because cells did not follow straight-line trajectories in most cases. Therefore, the range of PPFD experienced varied from cell to cell, depending on their motility and exploratory behavior (Fig. 5b). The widest range of PPFD experienced by a cell was 1304 µmol photons m^−2^ s^−1^ (cell n°16) and the smallest range was only 22 µmol photons m^−2^ s^−1^ (cell n°4), with an average change across cells of 476 ± 373 µmol photons m^−2^ s^−1^.

**Fig. 6:**
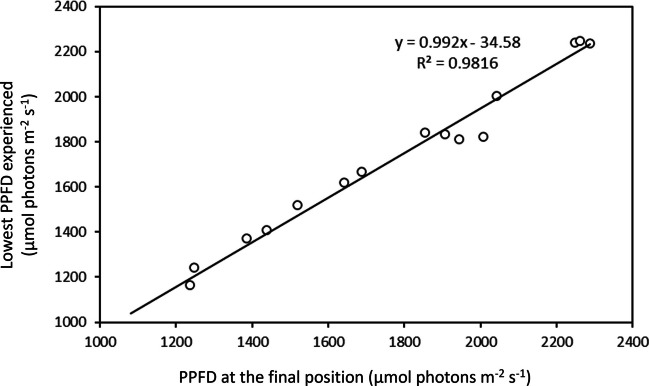
Linear relationship between the lowest photosynthetic photon flux density (PPFD) experienced by each individual cell during the 32-min exposure period and the PPFD at their final position by the end of the 32-min exposure period

A strong correlation was found between the PPFD experienced at the final position at the end of the exposure and the lowest PPFD experienced by each cell along the exposure (*r* = 0.991; *p* < 0.001; Fig. 6).

### Motility and Experienced Light Dose

**Fig. 7:**
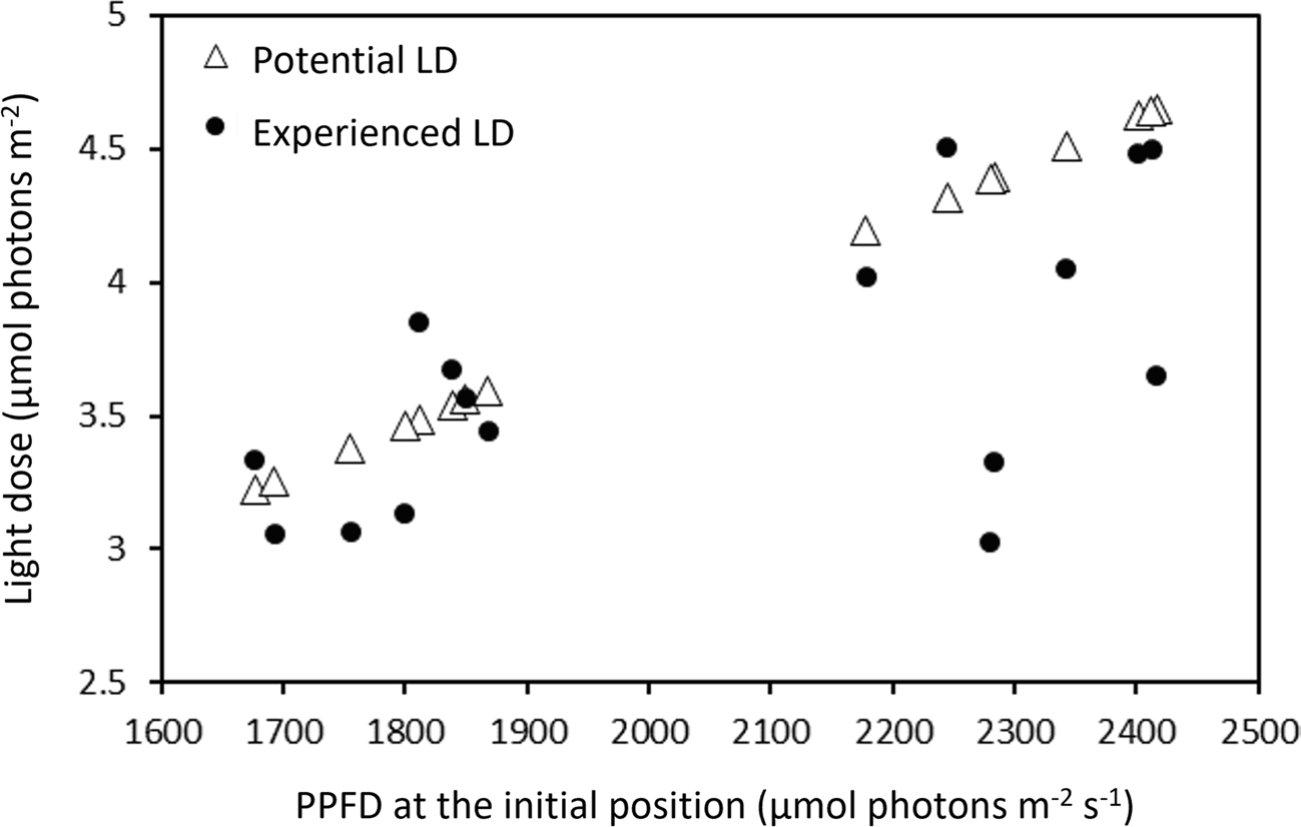
Difference between the cumulative light dose (LD) experienced by the cells during the experiment (using motility; experienced LD), and the potential cumulative light dose if cells would have remained immotile at their initial position (potential LD). The potential LDs were calculated based on the photosynthetic photon flux density (PPFD) at the initial position of cells, extrapolated over the 32-min exposure period

The motility behavior of the cells led them to experience a cumulative light dose (experienced light dose, E-LD) markedly different from the cumulative light dose they would have experienced without moving (potential light dose, P-LD). Among the 8 cells initially located in the 2000–2500 µmol photons m^−2^ s^−1^ region, 7 experienced an E-LD 3 to 31% lower than the P-LD (Fig. 7); only one cell showed a marginally higher E-LD (+ 4.4%). In comparison, for the cells initially located in the 1500–2000 µmol photons m^−2^ s^−1^ region, half of them experienced a higher E-LD (between + 0.1 and + 10.5%) while the other half experienced a lower E-LD (between − 4.2 and 9.4%) than the P-LD (Fig. 7). The two cells that did not move during the entire experiment showed a E-LD of 1.82 and 2.17 mol photons m^−2^.

### Effective PSII Quantum Yield

**Fig. 8:**
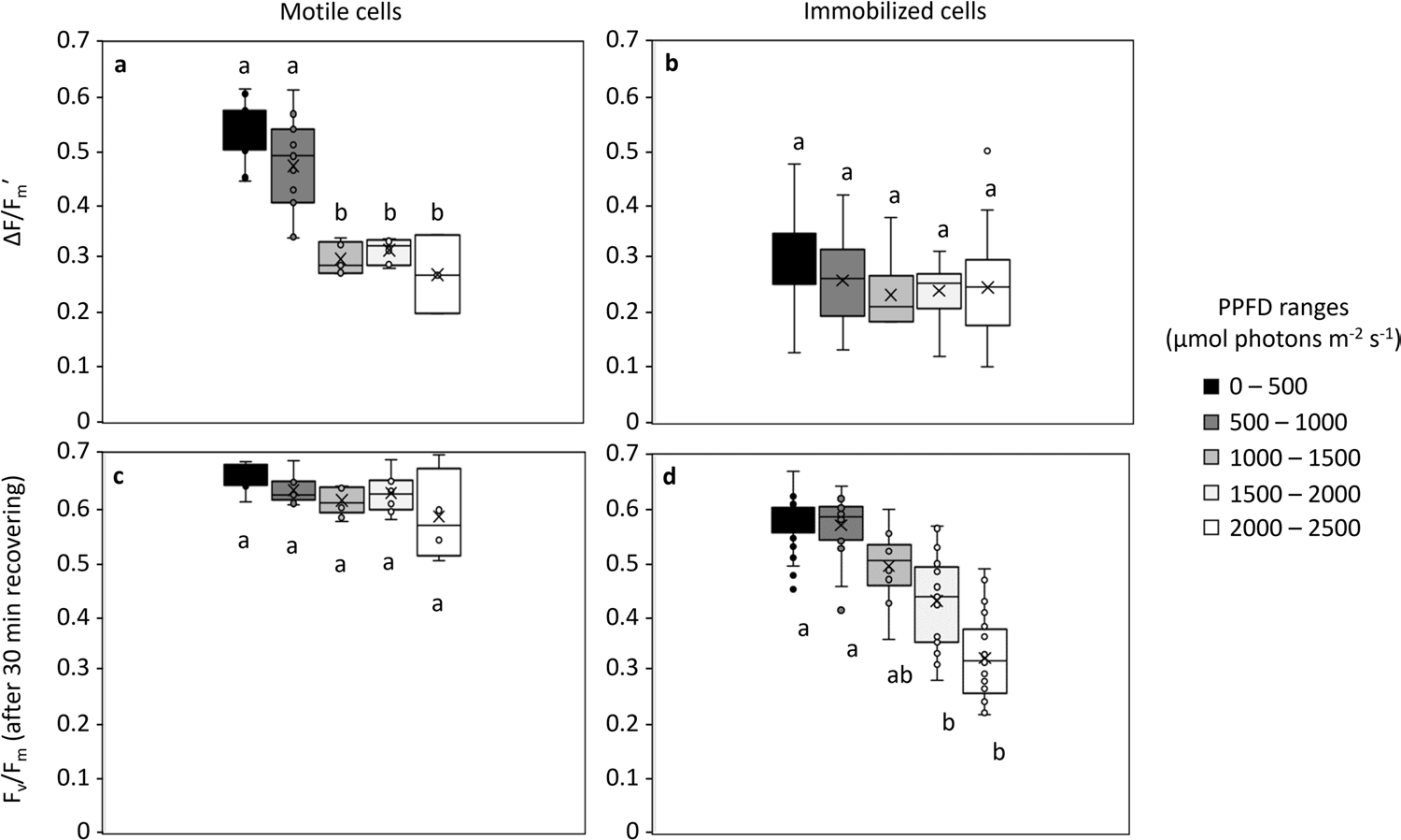
Effective quantum yield of PSII (Δ*F*/*F*_m_ʹ) at the end of the light exposure (**a**, **b**) and maximal quantum yield of PSII (*F*_v_/*F*_m_) after 30 min of recovery under darkness (**c**, **d**) for motile cells (**a**, **c**), and immobilized cells treated with latrunculin A (**b**, **d**). The values, measured for each individual cell located over the entire photosynthetic photon flux density (PPFD) gradient, were grouped in PPFD ranges (intervals of 500 µmol photons m^−2^ s.^−1^) according to their positions at the end of light exposure period. Lowercase letters refer to statistical differences between classes obtained after one-way ANOVA or Kruskal Wallis tests followed by pairwise multiple comparison procedures (Dunn’s method or TukeyHSD tests)

After the 32 min of exposure under the light microgradient, Δ*F*/*F*_m_ʹ was significantly higher for cells finally located under 0–1000 µmol photons m^−2^ s^−1^, reaching on average 0.505 ± 0.06 (*n* = 22), while cells finally located under 1000–2500 µmol photons m^−2^ s^−1^ showed on average 0.301 ± 0.03 (*n* = 14; Fig. 8a). Across the population of 18 cells monitored over the entire experiment, no significant correlations were found between Δ*F*/*F*_m_′ and the light parameters, including the initial (*p* = 0.2), final (*p* = 0.6), minimum (*p* = 0.6), or maximum (*p* = 0.2) PPFD experienced, the range of PPFD experienced (*p* = 0.4) nor E-LD (*p* = 0.4). Δ*F*/*F*_m_ʹ showed a positive correlation with *Y*(NPQ) that approached significance (*p* = 0.06). Likewise, a positive correlation between Y(NPQ) and the final PPFD experienced approached significance (*p* = 0.07).

When applying the motility inhibitor Lat A, and after 32 min of exposure under the light microgradient, Δ*F*/*F*_m_ʹ averaged 0.266 ± 0.08 (*n* = 101), without significant differences between the cells located in different PPFD ranges (aov; *p* > 0.05; Fig. 8b).

For the control experiments, 32-min exposure to flat LL conditions did not result in a significant difference between Δ*F*/*F*_m_ʹ (on average 0.635 ± 0.07; *n* = 27) and the initial *F*_v_/*F*_m_ values. On the other hand, after 32-min exposure to flat HL conditions, all cells located in the view field showed significantly lower Δ*F*/*F*_m_ʹ values (on average 0.392 ± 0.09; *n* = 43) in comparison to the initial *F*_v_/*F*_m_ values.

### PSII Quantum Yield Recovery

**Fig. 9:**
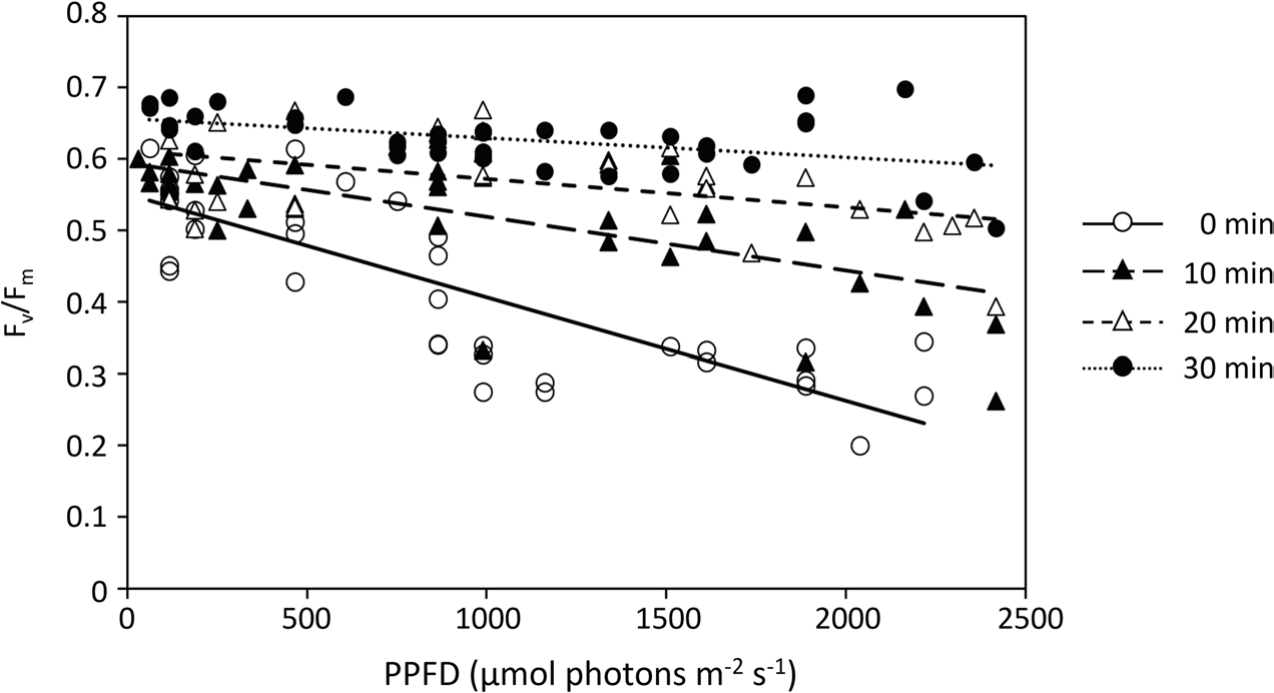
Recovery of the maximum quantum yield of PSII (*F*_v_/*F*_m_) of individual cells over the dark period (0–30 min) following the 32-min light exposure, as a function of the photosynthetic photon flux densities (PPFD) corresponding to their positions at the end of the light exposure period. At time 0, the values measured correspond to the effective quantum yield of PSII (Δ*F*/*F*_m_ʹ) as measurements were done directly after light exposure without dark relaxation

During the post-exposure dark period of the main experiment, all the cells gradually recovered, as represented by the progressive increase from Δ*F*/*F*_m_ʹ to *F*_v_/*F*_m_ after 30 min (Fig. 9). The recovery time was positively correlated with the last PPFD experienced. For cells located under 1000–1500 µmol photons m^−2^ s^−1^ at the end of the exposure period, a period of 19 min was required for a complete recovery (*F*_v_/*F*_m_ of 0.6), while periods of 22 and 32 min were required for cells located under 1500–2000 and 2000–2500 µmol photons m^−2^ s^−1^, respectively.

All the cells present in the light microgradient, including the 18 monitored cells, completely recovered by the end of the post-illumination period (Fig. 8c), reaching *F*_v_/*F*_m_ values of on average 0.622 ± 0.05 with no significant differences between the cells located in different PPFD ranges (kw; *p* = 0.05). Similar results were observed for cells exposed to LL and HL conditions that completely recovered after 30 min in the dark, reaching *F*_v_/*F*_m_ values of on average 0.650 ± 0.07 (*n* = 33) and 0.643 ± 0.09 (*n* = 46), respectively.

When using the motility inhibitor Lat A, complete recovery was observed only for the immobilized cells under 0–1000 µmol photons m^−2^ s^−1^, reaching 0.568 ± 0.05 after 30 min (Fig. 8d). In comparison, after 30 min in the dark, an incomplete recovery was observed for immobilized cells located on the upper part of the light microgradient, showing *F*_v_/*F*_m_ of on average 0.477 ± 0.07, 0.434 ± 0.09, and 0.325 ± 0.07, respectively, for 1000–1500, 1500–2000, and 2000–2500 µmol photons m^−2^ s^−1^ (Fig. 8d).

## Discussion

The novel approach developed in this work allows study of the photobehavior and photophysiology of individual motile photoautotroph cells under light microgradients. This set up allows analysis of cellular behavior in response to spatial heterogeneity in light microgradient by tracking individual cells over time and supports quantitation of the cumulative light dose received by individual cells, leading to the characterization of the experienced light environment (constructed light niche [22]). Furthermore, by allowing the combination of motility and photophysiology responses to light, our study provides new experimental evidence supporting the behavioral photoprotective hypothesis, showing that benthic diatom cells exposed to high light levels use motility to reduce their experienced light dose, avoiding photoinhibition.

### Single-Cell Motile Photobehavior

The motility behavior of epipelic diatoms is species-specific [33, 53] and associated with species-specific sensitivities to light wavelength and intensity [14, 38, 49, 54], and with species-specific EPS secretion patterns during gliding, related to the form of their raphe [42, 55]. Different diatom species present different schemes of motility including apical, transapical, and polar gliding, interrupted by back-and-forth movement and by complex motion sequences such as horizontal rotation and vertical pivoting [53, 56]. In the present study, in a mono-specific culture of *Craspedostauros britannicus*, several different movement patterns were observed (Online Resource 3 and 4), highlighting the large variability in motility behavior among cells of the same population. All the observed types of cellular movements seemed to contribute towards adjustment of the light dose experienced by the cell, with the potential to optimize photosynthesis.

Many environmental factors influence diatom motility, including light intensity and light quality, temperature, salinity, pH, tidal phase, disturbance, desiccation, signaling molecules, and nutrients [11, 57–61] with the potential to trigger species-specific and potentially cell-specific reactions [14]. Considering the small dimensions and the channels of the microfluidic chips used here, good homogenization of the cell suspension inside the channel achieved through injection, and the low cell density that was used, it can be assumed that abiotic conditions, aside from irradiance, remained nearly constant across cells during the relatively short duration of the experiments (i.e., 32 min) and that the light gradient was the main external factor affecting cell motility.

A common behavior of the tracked individual diatom cells was that, following light exposure, there was a 5-min lag before initiation of light-directed movements. This could be interpreted as resulting from a preliminary time interval needed by the cells to perceive the heterogeneity of the light microenvironment in their close surroundings. This 5-min lag period before the activation of motility is in accordance with previous work suggesting that diatom movement is primarily a response to increasing light dose [49]. By considering the time before activation of the motility, a cumulative light dose higher than 0.45 mol photons m^−2^ (1500 µmol photons m^−2^ s^−1^ for 5 min) could lead to a motility response. However, two cells initially located, respectively, at 948 or 1126 µmol photons m^−2^ s^−1^ did not show active movements during the entire experiment, despite an experienced light reaching 0.45 mol photons m^−2^ in less than 10 min (6 min 40 s and 7 min 55 s, respectively). The main difference between the cells that showed an active motility or not was the initial PPFD to which they were exposed. Thus, we suggest that the rate at which the light dose accumulates may have a larger impact on the induction of motility than the overall cumulative light dose. The difference in rate of PPFD increase was suggested to explain different photoprotective responses of a coastal phytoplanktonic diatom [62]. Therefore, we suggest that a light dose that accumulates at a higher (or lower) rate than the rate experienced under optimal light intensities for photosynthesis would result in an activation of the motility to reach more optimal intensities. In our study, the optimal light intensities were estimated through the range of *E*_opt_ values, suggesting that an increase of the light dose at a rate higher (or lower) than the rate experienced under 1084.3 ± 223.4 µmol photons m^−2^ per second (corresponding to *E*_opt_) would induce motility. Unlike the primary response to an increasing light dose, which would not induce motility at too low light levels, a low rate of increasing light dose could explain the activation of motility at low light levels. The influence of the rate at which the light dose accumulates is an additional factor to consider, besides the ones already assumed to induce diatom motility (for review, see [58]).

### Motility Is Used to Adjust the Experienced Light Environment

A main aspect of the behavioral photoprotective hypothesis is the use of motility to alleviate exposure to excessive light levels, potentially capable of photodamaging effects [25, 41]. The results of this study support this idea, showing that, when exposed to excessive light levels, diatom cells use motility to reduce their experienced light dose.

The first supporting evidence is that the cells that started the exposure period under high PPFD, tended to be found, at the end of the exposure, under the lowest PPFD they had experienced during the exposure period. While this result undoubtedly shows that diatom cells use motility to regulate their experienced light dose, it does not directly support the hypothesis that cells would move to avoid PPFD above their photosynthesis optimum, *E*_opt_. The optimal light intensity estimated from SSLCs for the culture used in this study was 1084.3 ± 223.4 µmol photons m^−2^ s^−1^, but, at the end of the light exposure period, 70% of the cells were found located in the light microgradient region above 1500 µmol photons m^−2^ s^−1^. The fact that cells were located at their individual lowest PPFD experienced, yet still higher than their estimated *E*_opt_, suggests that phototaxis is not as efficiently directed towards *E*_opt_ as hypothesized.

Photobehavior relies upon the perception of light by photosensitive molecules that act as light sensors [63]. Despite the increasing knowledge on mechanisms controlling diatom light sensing [64], it is not known across what distance diatoms can perceive a change in PPFD. It was shown that the direction of diatom movement is strongly correlated with the light intensity that the tips of cells were exposed to, with positive or negative phototaxis depending on the intensity received on each tip [54, 65, 66]. It can thus be assumed that light changes are distinguishable across the scale of individual cells. This suggests that pennate diatom cells might need to actually experience different PPFD values across the span of their cell before being able to re-orient their movement. This could explain our results since cells might need a longer time than used in this study to explore the light environment in order to detect and reach a light region optimal for photosynthesis. Thus, the motility response to light gradients would result from a mix of exploratory and light-oriented movements. Motility would be used to explore the light environment, after which light-oriented movements would be based on the experienced light intensities across the length of a cell.

The second evidence indicating that diatoms use motility to regulate their experienced light environment is the observation that cells, initially exposed to high PPFD and that moved along the light microgradient, experienced a cumulative light dose lower than the potential light dose they would have received if they did not move. The cumulative light doses for motile cells were up to 31% lower than their potential light doses without movement from initial position, the difference being positively correlated with the PPFD to which they were exposed at their initial position. This result confirms that motility enables diatoms to actively choose their light environment in terms of mean exposures and variability of light dose received, including in this case the duration of time under exposure to high PPFD [22].

By assuming that epipelic diatoms use motility to optimize light level for photosynthesis, one could argue that the cells that did not move were located at a light level which did not require a supplementary energy expense to be optimized through motility. The cost of releasing nano-polymeric fibers to glide [67] was estimated at 0.12 pJ for a benthic diatom cell to glide on 400 µm [68], and although this cost was estimated to be very low relative to their daily net photosynthetic production, it is still an expense which should be minimized if not necessary. This suggests that the intensities of 948–1126 µmol photons m^−2^ s^−1^ at the initial location of those two cells were neither too low for optimal photosynthesis nor physiologically harmful for this species/cells. This assumption is supported by the *E*_opt_ values of the population of 1084.3 ± 223.4 µmol photons m^−2^ s^−1^ and particularly by the complete recovery of the quantum yield of fluorescence for these two cells during dark relaxation subsequent to the 32-min light exposure period. We also suggest that the two cells that did not move experienced an optimal rate of light dose accumulation which resulted in a cumulative light dose representative of the cells light niche [22].

### Motility Has a Photoprotection Role

The exposure of photosynthetic organisms to high light levels usually results in inhibition of PSII activity, with consequent negative impacts on photosynthesis and growth [69–71]. The magnitude of photoinhibition is commonly detected and quantified based on the recovery kinetics of the non-photochemical chlorophyll fluorescence quenching (NPQ) following a light stress period [70, 72, 73]. As inhibition of PSII results in a lower ability to transfer electrons, recovery kinetics can also be measured by monitoring the changes in *F*_v_/*F*_m_ over time [74] and, in the case of benthic diatoms, decrease in Δ*F*/*F*_m_ʹ has been also used [39].

In diatoms, NPQ is composed of two main components, high-energy state quenching (*q*_E_), and photoinhibitory quenching (*q*_I_) [75, 76]. *q*_E_ and *q*_I_ components are identified and distinguished from each other by their different relaxation times and result from different physiological mechanisms. The *q*_E_ component is rapidly reversible over the first 10–30 min after light stress. *q*_E_ results in dissipation of excitation energy as heat, in response to energization of the thylakoid membrane, which creates a proton gradient (ΔpH) [77]. *q*_E_ requires the xanthophyll cycle [78]. *q*_E_ is considered important in photoprotecting PSII against net photoinhibition [39, 72, 79]. On the other hand, *q*_I_ is slowly reversible over a time scale of hours, as it is related to the photoinactivation of the PSII D1 protein [80–82], with relaxation through subsequent repair of PSII, requiring de novo synthesis and activation of a new protein [70, 83, 84]. Even for species with a rapid D1 turnover repair cycle, the relaxation of *q*_I_ process is generally longer than for *q*_E_ [80, 85, 86]. However, it is important to consider recent studies highlighting that *q*_I_ is not the only slowly relaxing NPQ process and that it can overlap with other types of NPQ involving sustained content of de-epoxidized xanthophylls zeaxanthin or diatoxanthin [87, 88].

A unique aspect of the approach of the present study was the possibility to concurrently measure cell motility and the consequent photophysiological effects, to understand whether and how much the motility of individual cells alleviates the *F*_v_/*F*_m_ decrease, most likely linked with PSII inactivation under high-light levels. Despite the complexity and intra-populational variability of the motility behavior observed at the cellular scale, the results showed that cellular motility contributed to minimize high light-induced decrease in photosynthetic efficiency, supporting the photoprotective role of photobehavior. This comes from the comparison of the *F*_v_/*F*_m_ recovery by freely motile (untreated) and motility-inhibited (treated with Lat A) cells. In untreated cells, a complete recovery was observed in virtually all cases, with post-recovery *F*_v_/*F*_m_ values not significantly different than the pre-stress ones, indicating that slowly reversible NPQ processes were not activated by the cells that were able to move. In contrast, cells treated with the motility inhibitor Lat A, showed post-recovery *F*_v_/*F*_m_ values significantly lower than the pre-stress ones, especially when exposed to PPFD higher than 1000 µmol photons m^−2^ s^−1^. The Lat A does not itself significantly affect photosynthetic processes over periods similar to those used in this study [8, 39, 46, 49]. Thus, the much slower recovery observed in *F*_v_/*F*_m_ of Lat A-treated cells, in comparison with untreated ones, can be considered to be mainly due to their inability to move within the light gradient. These results thus indicate that physiological photoprotective processes alone are not sufficient to prevent photoinhibition in benthic diatoms. Full photoprotection can only be secured by cellular motility-based regulation of light exposure, confirming the photoprotective nature of photobehavior. Under natural conditions, the photoprotective response to high light is thus likely to rely on the interactive contributions of rapidly reversible NPQ processes and light-directed motility, acting at overlapping timescales [26].

### Advantages, Limitations, and Further Applications for the Developed Setup

The experimental setup developed in this study represents a significant improvement in the capacity to study the in vitro photobehavior of motile microorganisms. In particular, it allowed us to address and test fundamental assumptions made in the last decades on the photoprotective role of motility on the photosynthetic performances of the motile benthic diatoms.

A major improvement consists in the generation of ecophysiologically realistic light microgradients over millimeter scales. Up to now, studies of motile benthic microalgae photobehavior have been based on observations under homogeneous light fields [89] or using unrealistically wide light gradients spanning centimeters [41]. Our setup design is able to produce light gradients of ~ 2 mm in length and covering PPFD from 0 to above 2000 µmol photons m^−2^ s^−1^ realistic with in situ light environment at the sediment surface [46]. Although this scale does not match the dimensions of the vertical profiles of diatom accumulation observed in the finest sediments [9], it can still be considered as a significant step forward, especially when considering the light attenuation depths in the intertidal sediments from 1 mm in mud to 3 mm in sand [8] and considering that microphytobenthos (MPB) biofilms are most productive in sediments with a sand-mud mixture where the photic zone is ca. 2 mm [40]. Although the developed setup allowed for the detailed study of the motility and photosynthetic responses to high light, there are still technical difficulties in studying cells in less illuminated regions of the gradient at the same measurement repetition frequency as those in the higher illuminated regions. However, the observation of cells in the lower light region could be overcome by decreasing the highest light level applied to the gradient.

A limitation of the present approach is that the experimental conditions do not fully represent the conditions experienced within natural sedimentary biofilms. In the present setup, the cells exclusively move along a horizontal plane, while in a biofilm, the variation in light availability is mainly vertical. In addition, cells are isolated from the heterogeneity of the sedimentary environment, including other chemical gradients, organisms, and sedimentary particles bonded with exopolysaccharides [58, 90]. However, the set-up was designed to precisely test the long-lasting hypothesis of the photoprotective behavioral motility. Future challenges will be to test this hypothesis in more complex environment, which include the synergetic effect of light microgradient with the other chemical microgradients of the sediment matrix.

The use of microfluidic chips provided a number of advantages for studying pennate diatoms at the cellular scale. The linear channel effectively limited cell movements to the main axis of the light gradient, while retaining dimensions adequate for diatoms. There is negligible light attenuation by the chip, enabling illumination of the cells with a wide range of light, including the measuring and saturating pulses of the microscopy-PAM fluorometer. As off the shelf components, the microfluidic chips are inexpensive and readily available, providing reproducible conditions. A problematic aspect is the length of the channel, as it is longer than the length of the applied light gradient, enabling cells to move to regions outside the microscope view field, which include the light microgradient, impeding maintenance of a stable number of cells within the experiment.

The use of the microscopy-imaging-PAM fluorometer was efficient for assessing the photophysiology of individual diatom cells distributed along the light microgradient. The use of this instrument was particularly advantageous considering that, unlike the case of the determination of the cell positions, which was difficult on the darkest side of the light gradient (see above), it was possible to make photophysiological measurements across the entire gradient. The main inherent limitation was the need to use a 20 × objective for fluorescence measurements, which required multiple measurements to cover the entire gradient.

Inhibition of diatom mobility was effectively achieved through the utilization of Lat A, chosen primarily for its easy implementation within the experimental design and lack of impact on photosynthetic processes [8, 39, 46, 49]. However, this must be carefully considered because environmental factors affecting motility can interact with chemical interventions [91] with unobservable consequences. Alternative approaches restricting diatom movement can also be explored, thus mitigating the potential impact of chemical intervention. Strategies such as encapsulating diatoms within a gel matrix [92] or incorporating them into alginate beads [93] represent viable alternatives worth considering. Although these methods represent promising alternatives to limit the movement of diatoms without the use of chemicals, they are virtually impossible to apply to the microfluidic chips used in this study.

In this study, we present the results of a single experiment, representative of the outcomes of several similar experiments performed. Presenting multiple experiments introduces additional sources of variability, such as variation in environmental conditions and cell populations, which could potentially confound the interpretation of results. We chose this approach to provide a more robust representation of the inherent variability in cellular responses to the light microgradient. The results were deemed sufficiently pertinent for capturing a representative spectrum of behavioral and physiological responses.

The present work centered on testing the photoprotective role of the cellular motility in benthic pennate diatoms. Only one species of pennate diatom was studied, as this work primarily aimed to serve as a proof of concept. The results obtained with *Craspedostauros britannicus* can now be complemented with similar studies on other diatom samples, presenting different motility and photophysiological characteristics, either single or mixed species grown in culture under changing growing conditions, or natural MPB communities, to deepen our knowledge on the ecologically and evolutionary important role of the diatom phototaxis. Although improvements are feasible, the presented setup is also readily usable with other microorganisms for which light represents a forcing environmental factor, including motile and non-motile, planktonic, and benthic growth forms. The setup is also easily adaptable to more specific or in-depth studies, e.g., on photoreceptors, for which the applied light spectrum could be modified to generate light conditions representative of particular habitats (i.e., subtidal versus intertidal sediments for instance).

### Supplementary Information

Below is the link to the electronic supplementary material.

Supplementary file1 (PDF 854 KB)

## Data Availability

No datasets were generated or analyzed during the current study.
