# Supplementary material for: The Photoprotective Behavior of a Motile Benthic Diatom as Elucidated from the Interplay Between Cell Motility and Physiological Responses to a Light Microgradient Using a Novel Experimental Setup
Source: Microb Ecol. 2024 Feb 13;87:40. doi: 10.1007/s00248-024-02354-7 (PMC10864569; doi:10.1007/s00248-024-02354-7)
Supplement: Supplementary file 1 [file 248_2024_2354_MOESM1_ESM.pdf]

Supplementary Information

The photoprotective behavior of a motile benthic diatom as elucidated from the interplay between cell motility and physiological responses to a light microgradient using a novel experimental setup

Microbial Ecology

Jérôme Morelle[1]*, Alexandra Bastos[1], Silja Frankenbach[1], Jörg C. Frommlet[1], Douglas A. Campbell[2], Johann Lavaud[3], João Serôdio[1]

[1]CESAM – Centre for Environmental and Marine Studies and Department of Biology, University of Aveiro, Campus de Santiago, 3810-193 Aveiro, Portugal

[2]Biology Department, Mount Allison University, Sackville, NB, Canada

[3]LEMAR - Laboratory of Marine Environmental Sciences, UMR 6539 CNRS, Univ Brest, Ifremer, IRD, Institut Universitaire Européen de la Mer, Technopôle Brest-Iroise, Plouzané, France

*Corresponding author: jerome.morelle@ua.pt ; Orcid: 0000-0003-2167-818X

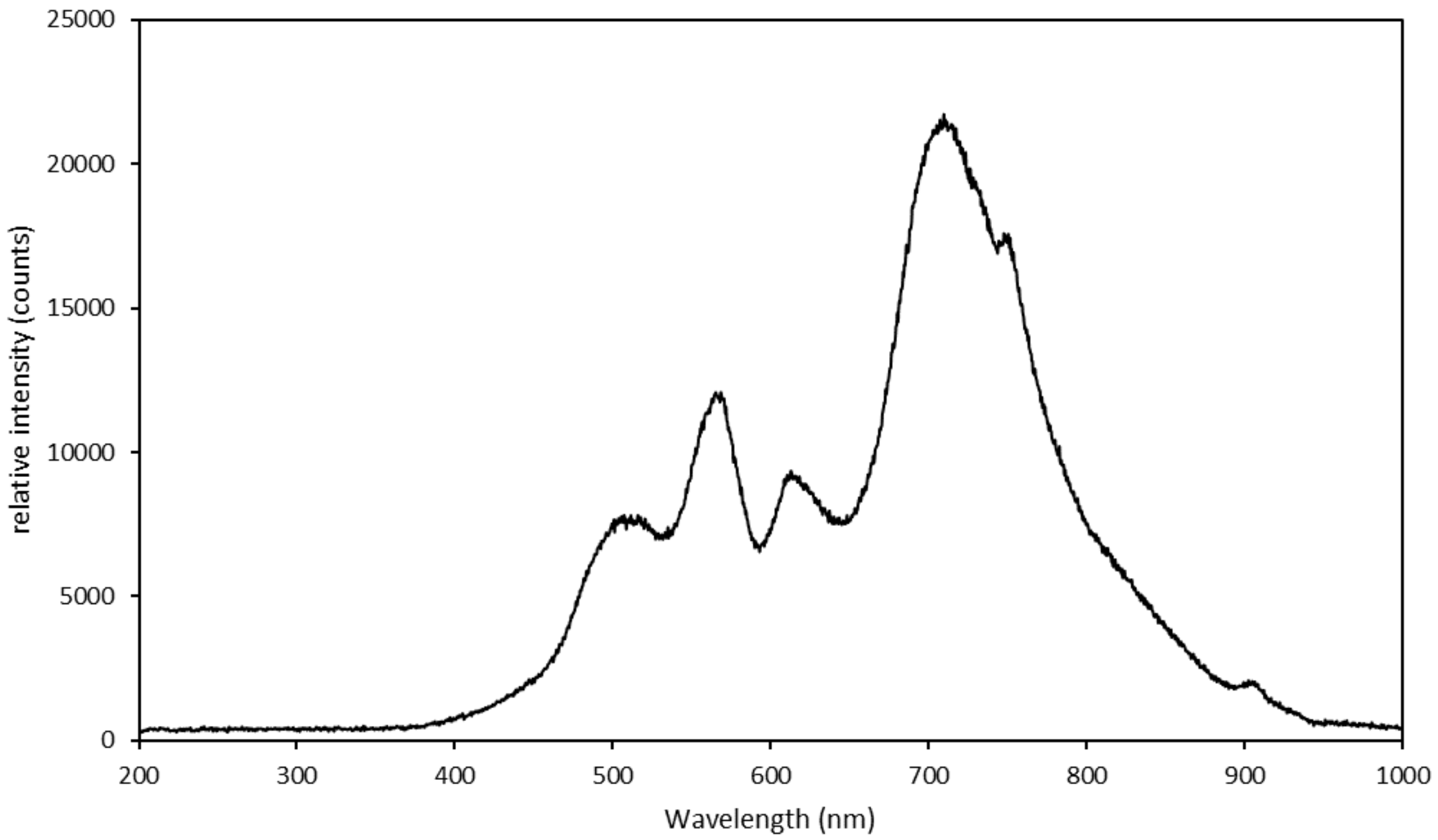

Online Resource 1. Spectrum of the light applied during exposure. This incident light was provided by the microscope's halogen lamp, filtered by a blue filter (1101001900355; Motic Deutschland GmbH, Wetzlar, Germany), to balance the blue vs red regions of the visible spectrum, and an UV/IR cut-off filter 390-690 nm (49-809, Edmund Optics Ltd, York, UK) to eliminate thermal infrared wavelengths and limit heating of the samples.

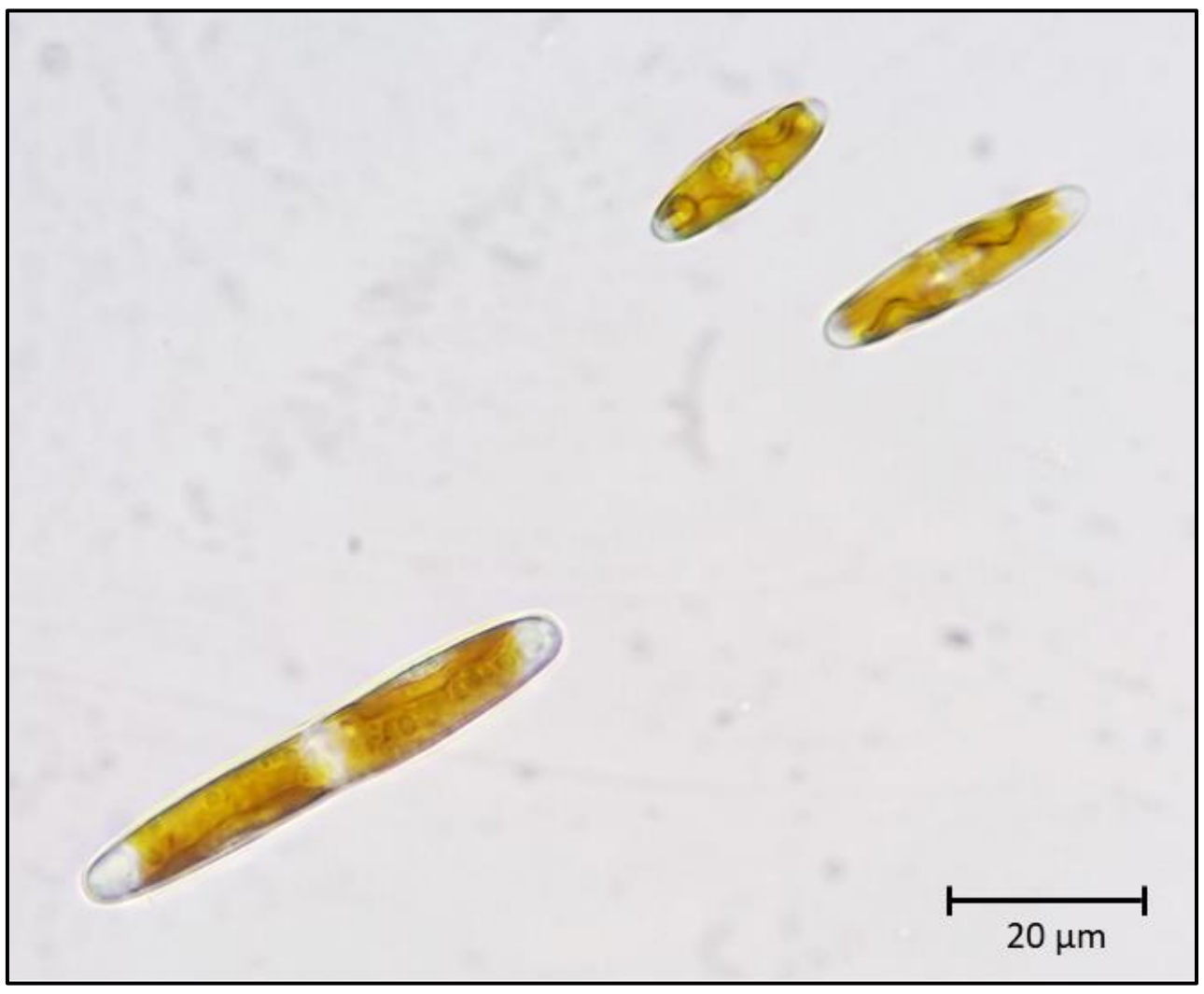

Online Resource 2. Micrograph of the species used in this study (*Craspedostauros britannicus*). The image shows the three different size classes of the cells observed in experimental culture from the smallest to the biggest cell size: 20, 30, and 55 µm length.

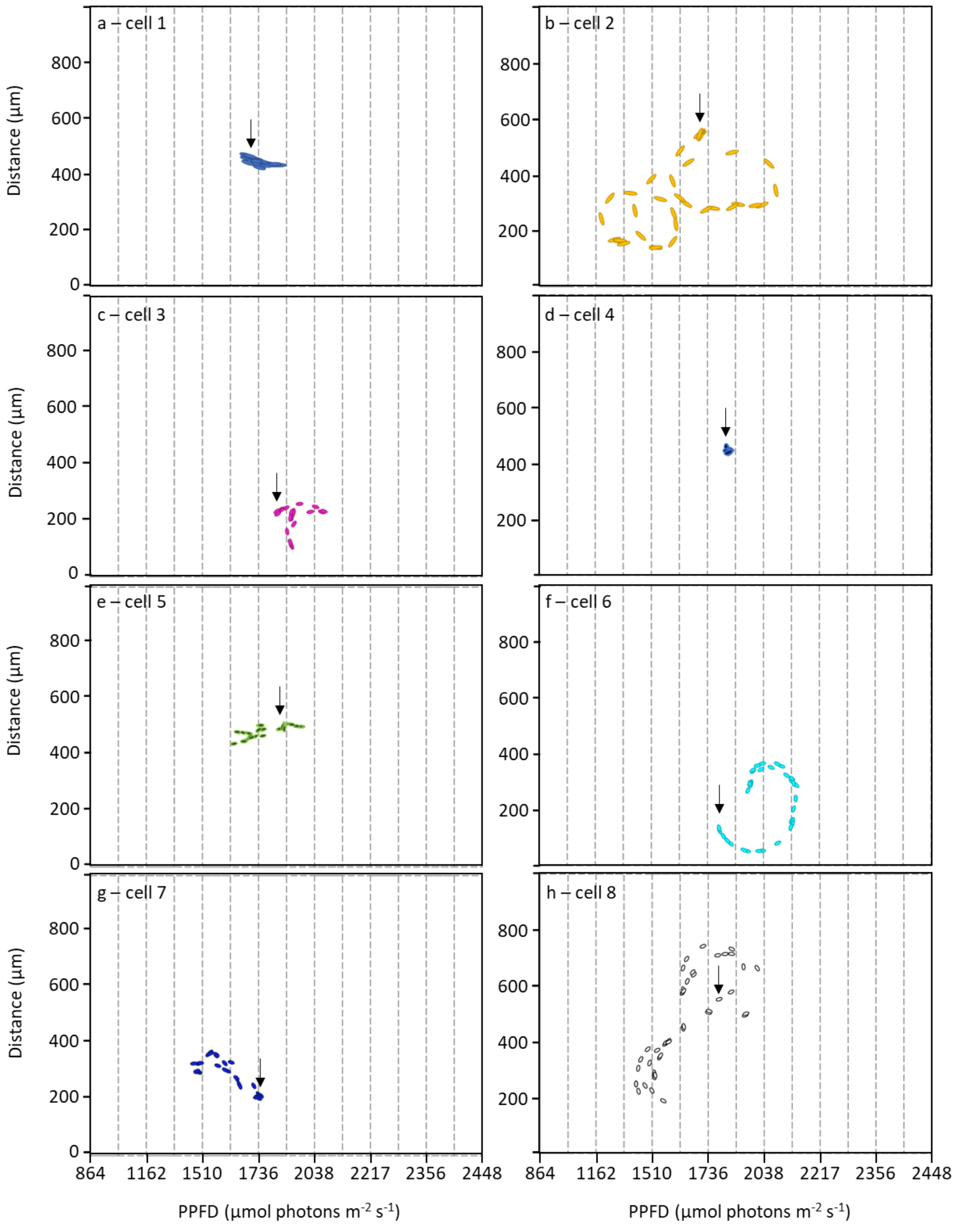

Online Resource 3. Variations of cell positions within the channel of the microfluidic chip initially exposed to 1500 – 2000 µmol photons m^{-2} s^{-1}. The X-axis shows a part of the gradient in photosynthetic photon flux density (PPFD) measured every 100 µm as indicated by the overlaid vertical dashed lines. The Y-axis represents the width of the area within the channel in which the PPFD is homogeneous. Positions were recorded every minute over a 32-min light exposure. The black arrow marks the initial position of each cell.

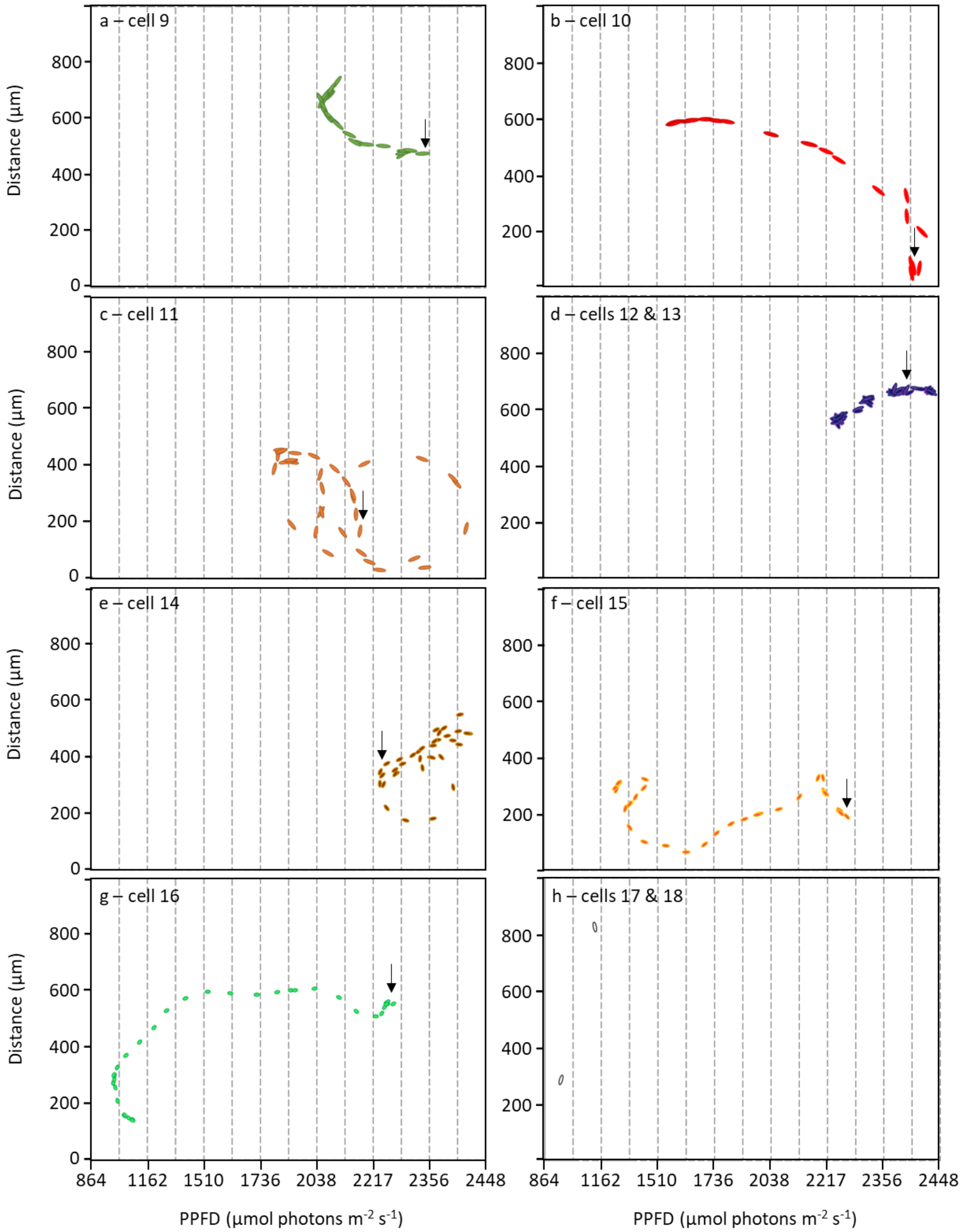

Online Resource 4. Variations of cell positions within the channel of the microfluidic chip initially exposed to 2000 – 2500 µmol photons m^{-2} s^{-1}. The X-axis shows a part of the gradient in photosynthetic photon flux density (PPFD) measured every 100 µm as indicated by the overlaid vertical dashed lines. The Y-axis represents the width of the area within the channel in which the PPFD is homogeneous. Positions were recorded every minute over a 32-min light exposure. The black arrow marks the initial position of each cell. Cells 12 & 13 (d) were presented together as, despite different behaviors, they were stuck over each other and stayed superimposed during the experiment. Cells 17 & 18 (h) did not move over the exposure period.
